# Promise and pitfalls of viscoelastic testing for assessing blood hemostasis in ultraendurance sports

**DOI:** 10.1016/j.rpth.2025.103011

**Published:** 2025-08-13

**Authors:** Apostolos Z. Skouras, Panagiotis Koulouvaris

**Affiliations:** Sports Excellence, 1^st^ Department of Orthopaedic Surgery, School of Medicine, National and Kapodistrian University of Athens, Athens, Greece

Ultraendurance races such as trail ultramarathons push human physiology to extremes, including the blood’s clotting (hemostatic) system. Although regular moderate exercise reduces thrombosis risk and promotes cardiovascular health, extreme efforts tell a more complex story. Events like 50 to 100 km trail runs create a cocktail of dehydration, inflammation, muscle damage, and exertional heat stress—all of which perturb hemostasis. Indeed, thromboembolic events have been documented in otherwise healthy marathoners [[1], [2], [3], [4]]. Prolonged running activates coagulation and, to a degree, the counterbalancing fibrinolysis that dissolves clots [1,5,6]. This “rebalanced” hemostatic state is often transient. However, some evidence suggests that the balance may tip unfavorably during recovery, with coagulation staying elevated while fibrinolysis wanes [7]. In practical terms, an ultramarathon finisher might display laboratory signs of hypercoagulability even hours postrace, akin to an acute inflammatory state.

This topic has gained relevance as endurance sports attract older and recreational participants. Master athletes (>40 years) now represent a growing proportion of ultramarathon fields [[8], [9], [10], [11]]. Age itself can augment prothrombotic tendencies; for example, older marathoners show higher baseline and postrace platelet activation (P-selectin levels) than younger runners [12]. Moreover, many ultra-athletes are recreational competitors whose training and recovery capacities differ from those of elite athletes. These trends raise pressing questions: Do extreme races provoke clinically significant clotting risks, especially in older or less-conditioned individuals? How do we assess and mitigate that risk without undermining exercise’s clear health benefits? Studying hemostasis in ultraendurance sport is therefore not a niche curiosity, but rather an important aspect of safeguarding a growing and diversifying athletic population.

The pilot study by Schobersberger et al. [13] addresses this challenge head-on at one of the world’s most challenging races, the 2023 World Mountain and Trail Running Championship (86.9 km, ∼6500 m ascent). Uniquely, they employed viscoelastic testing (VET; ClotPro), alongside standard plasma coagulation assays, in elite trail runners. VET assesses whole-blood clot formation and lysis in real time, offering a broader view of hemostasis; rather than measuring isolated factors in plasma, it captures the net effect of procoagulant and anticoagulant forces. In critical care, VET has proven valuable for detecting phenomena such as fibrinolysis shutdown. a potentially life-threatening impairment of clot breakdown that is seen in trauma or sepsis [14]. Applying such technology to sports settings opens new investigative and possibly clinical pathways.

To our knowledge, this is the first application of ClotPro VET in athletes under extreme physical stress. Seven World Championship competitors were sampled before and within ∼3 hours postrace. Standard laboratory tests tracked coagulation factors changes, while the VET device assessed clot dynamics, including a tissue plasminogen activator assay to challenge the fibrinolytic system. Despite the small sample size, the findings illuminate several hallmarks of exercise-induced hemostatic change. Immediately after the 86.9 km trail run, a shift toward a hypercoagulable, inflammation-associated profile was detected (eg, prolonged prothrombin time [PT] coupled with reduced levels of factors V, VII, and X). This might seem paradoxical—a longer PT usually reflects impaired clotting—but it was counterbalanced by a marked elevation in factor VIII, factor IX and other intrinsic pathway factors, as well as von Willebrand factor, leading to a significantly shortened activated partial thromboplastin time (aPTT). In other words, the intrinsic coagulation pathway was highly activated postrace. Importantly, the authors also presented intra-individual hemostatic changes, providing valuable insight into personal variability in response to extreme exertion. The runners’ fibrinolytic response showed mixed features: plasminogen levels dropped and α2-antiplasmin (a fibrinolysis inhibitor) rose, alongside a spike in D-dimer (a fibrin degradation product). Elevated D-dimer indicates that substantial clot formation and breakdown did occur. However, the concomitant rise in fibrinolysis inhibitors suggests the body was also throttling back on clot breakdown – an observation consistent with an inflammatory “fibrinolysis shutdown” pattern seen in critical illness.

Importantly, VET results supported a prothrombotic tilt with restrained fibrinolysis. Since fibrinogen and platelet effects increased fibrin clot polymerization, postrace fibrin clot firmness increased, while clot lysis decreased. In plain terms, the blood clots formed after the race were sturdier and a bit more resistant to dissolution. The changes observed were laboratory phenomena; none of the athletes had clinical thrombotic symptoms. Yet, the study underscores that an ultraendurance race triggers coagulation changes qualitatively similar to an acute inflammatory or hypercoagulable state, albeit in transient fashion (Figure) [1,5,6,13,15].

**Figure:**
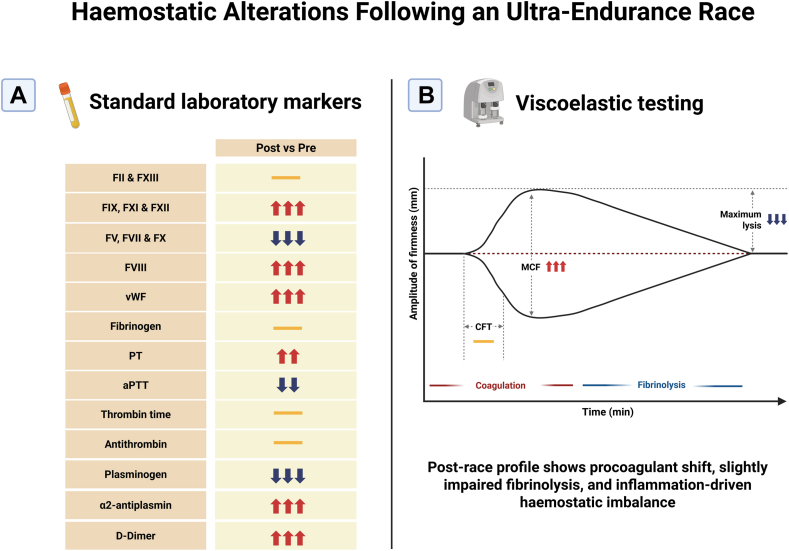
Hemostatic alterations after an ultraendurance race. Schematic summary of hemostatic changes observed before and after an 86.9 km trail ultramarathon. This figure is based on the findings of Schobersberger et al. [13], supported by related studies [1,5,6,15]. Panel A shows standard laboratory markers reflecting shifts in coagulation and fibrinolysis. Panel B reflects a viscoelastic profile indicating increased clot firmness and reduced fibrinolysis, consistent with a transient procoagulant, inflammation-associated state. Prolonged PT is represented as an increase in clotting time. Abbreviations: aPTT, activated partial thromboplastin time; CFT, clot formation time; FII, FIII, etc., factor II, factor III, etc.; PT, prothrombin time; MCF, maximum clot firmness; vWF, von Willebrand factor. Number of arrows denotes effect size: 2 for moderate, 3 for large; (–) indicates no or small nonsignificant change. Created in BioRender. https://biorender.com/2nge54h.

These findings are insightful, but this study has limitations that limit our interpretations. The small sample of elite athletes, along with race-specific factors (e.g. altitude, downhill terrain, temperate weather), limit statistical power and generalizability, particularly to recreational or older runners. Sex-based analysis was also not possible, despite known hormonal and hemorheological influences on coagulation. Timing of postrace blood draws is another concern. Samples were obtained “within 3 hours,” a window during which hemostatic markers can shift rapidly [7,16]. Another limitation involves the absence of direct platelet function testing. While ClotPro reflects overall clot firmness (to which platelets contribute), it cannot replace dedicated assays or biomarkers such as aggregometry, P-selectin, β-thromboglobulin, or platelet-derived microparticles. The importance of platelet assessment is supported by a prior study, which found that while coagulation was activated across marathon, triathlon, and cycling events, significant platelet activation occurred only in the running-intensive events, correlating with the greater clot risk in long-distance running [1]. By not measuring platelet activation or aggregation directly, the study leaves a gap in our understanding: we know the clots were “firmer,” but we do not know if platelets were hyper-reactive or paradoxically exhausted.

The findings of Schobersberger et al. [13] raise broader questions about how we assess and manage exercise-induced coagulation changes. How concerned should we be about post-ultramarathon hypercoagulability—is it a benign physiological adaptation or a real thrombotic risk? Although symptomatic venous thrombosis in endurance athletes is rare, it can occur under certain conditions, particularly when combined with dehydration, immobilization, or travel [12,17,18]. In fact, one study found that several marathoners had D-dimer levels >500 ng/mL (venous thromboembolism suspicion) the day after a race and a long-haul flight [12], suggesting a “second-hit” scenario may tip the balance toward thrombosis. Yet, our ability to identify who is at risk—and when—remains limited. Standard laboratory tests (eg, D-dimer, PT/activated partial thromboplastin time) are often too indirect or insensitive to guide individual risk, as they can be elevated by exercise without any pathology. This is where VET holds promise: by assessing clot formation and breakdown in real time, tools like ClotPro may help flag at-risk athletes. For example, this study used the ClotPro tissue plasminogen activator assay to detect fibrinolytic resistance (clot lysis impairment) that D-dimer measurements alone could not. In the future, VET could be used in research or event medical tents to identify athletes with hypercoagulable blood after extreme races.

That said, significant gaps in knowledge remain before VET can be meaningfully integrated into athlete care. We do not yet know what the normal reference range for thromboelastographic parameters is in exhausted but healthy athletes, nor what degree of anomaly correlates with clinical issues. While the pilot data suggest a “fibrinolysis shutdown” postrace, no thrombotic events occurred, so no risk threshold can be defined. Larger studies should correlate viscoelastic metrics with symptoms or imaging findings in the days after ultramarathons to identify predictive patterns (eg, low clot lysis or high clot firmness). Another gap is understanding the time course of recovery: How quickly does the hemostatic system normalize? Is there a rebound hyperfibrinolysis phase (“overshoot” of clot breakdown) after the initial shutdown, or do things simply return to baseline? For athletes who do serial ultraendurance events, does each bout impose cumulative stress on coagulation or do adaptive mechanisms develop? These questions are ripe for investigation.

From a sports medicine perspective, there is also interest in exploring preventive or mitigative strategies if a risky hemostatic profile is identified. Could simple strategies—aggressive hydration, cooling, gradual active recovery, compression garments, or timing of postrace travel—make a difference in normalizing coagulation faster? Preliminary evidence indicates compression socks may attenuate D-dimer increases during marathons [19], supporting mechanical interventions. Pharmacologic prophylaxis (eg, low-dose aspirin or anticoagulants) is not recommended for healthy athletes [20], but one could envision it being studied in those with extreme laboratory abnormalities or strong risk factors. Any such measures would have to carefully balance bleeding risk, of course, especially since ultra-athletes often already have muscle damage (and occasionally acute kidney injury or other issues) postrace. At present, we simply do not know enough to issue guidelines, highlighting the need for more data.

Encouragingly, the commentary’s title, “Promise and Pitfalls,” reflects exactly where we stand. The promise is that VET might become a powerful tool to illuminate the real-time coagulation status of athletes, much like heart rate monitors and lactate tests inform us about cardiovascular and metabolic status. It offers a chance to move beyond blunt measures and see the fine balance of clotting and bleeding in action. The pitfalls, however, include overinterpreting small studies, mistaking laboratory changes for pathology, and jumping to clinical use before evidence is ripe. We must remember that a hypercoagulable laboratory profile in a young athlete is not the same as that in a hospitalized patient; the implications could be vastly different.
